# Supplementary material for: Probiotic Bifidobacterium breve MCC1274 Protects against Oxidative Stress and Neuronal Lipid Droplet Formation via PLIN4 Gene Regulation
Source: Microorganisms. 2023 Mar 20;11(3):791. doi: 10.3390/microorganisms11030791 (PMC10052176; doi:10.3390/microorganisms11030791)
Supplement: Supplementary file 1 [file microorganisms-11-00791-s001.zip › microorganisms-2238966-supplementary.pdf]

microorganisms

Table S1. List of metabolites and internal standard.

Compounds	Abbreviation	Polarity	Precursor (m/z)	Product (m/z)	Collision energy (V)	CAS registry number
nicotinic acid	NA	Positive	124.05	78.042	21.47	59-67-6
nicotinamide	NAM	Positive	123.05	80.071	19.91	98-92-0
nicotinamide riboside	NR	Positive	255.093	123.071	10.77	1341-23-7
nicotinamide adenine dinucleotide	NAD+	Positive	664.071	427.97	26.06	53-84-9

Table S2. List of human gene primers used for PCR reactions.

Plin1	forward	5′-AGTCCAGGCCTGTGTGCTTTG-3′
	reverse	5′-GTGACTATGCAGGTGAAGGCAGTAA-3′
Plin2	forward	5′-TTCGCCTTTCGCTGCAGTC-3′
	reverse	5′-CCGAGTCACCACACTCTGCAAT-3′
Plin3	forward	5′-GCAAGCTTCGAGCCACCAA-3′
	reverse	5′-GGTTCCAGCTGAGCCACATC-3′
Plin4	forward	5′-GGAGCTGCAACCTTCGGAAA-3′
	reverse	5`-ATCCTTGGCCCTGGACATCT-3′
Plin5	forward	5′-TGCTGCTCAGCCTGCCATAC-3′
	reverse	5′-AGGACCTTTATTCTGGAGGCAAATC-3′
GAPDH	forward	5′-GCACCGTCAAGGCTGAGAAC-3′
	reverse	5′-TGGTGAAGACGCCAGTGGA-3′
IL-6	forward	5′-AAGCCAGAGCTGTGCAGATGAGTA-3′
	reverse	5′-TGTCCTGCAGCCACTGGTTC-3′

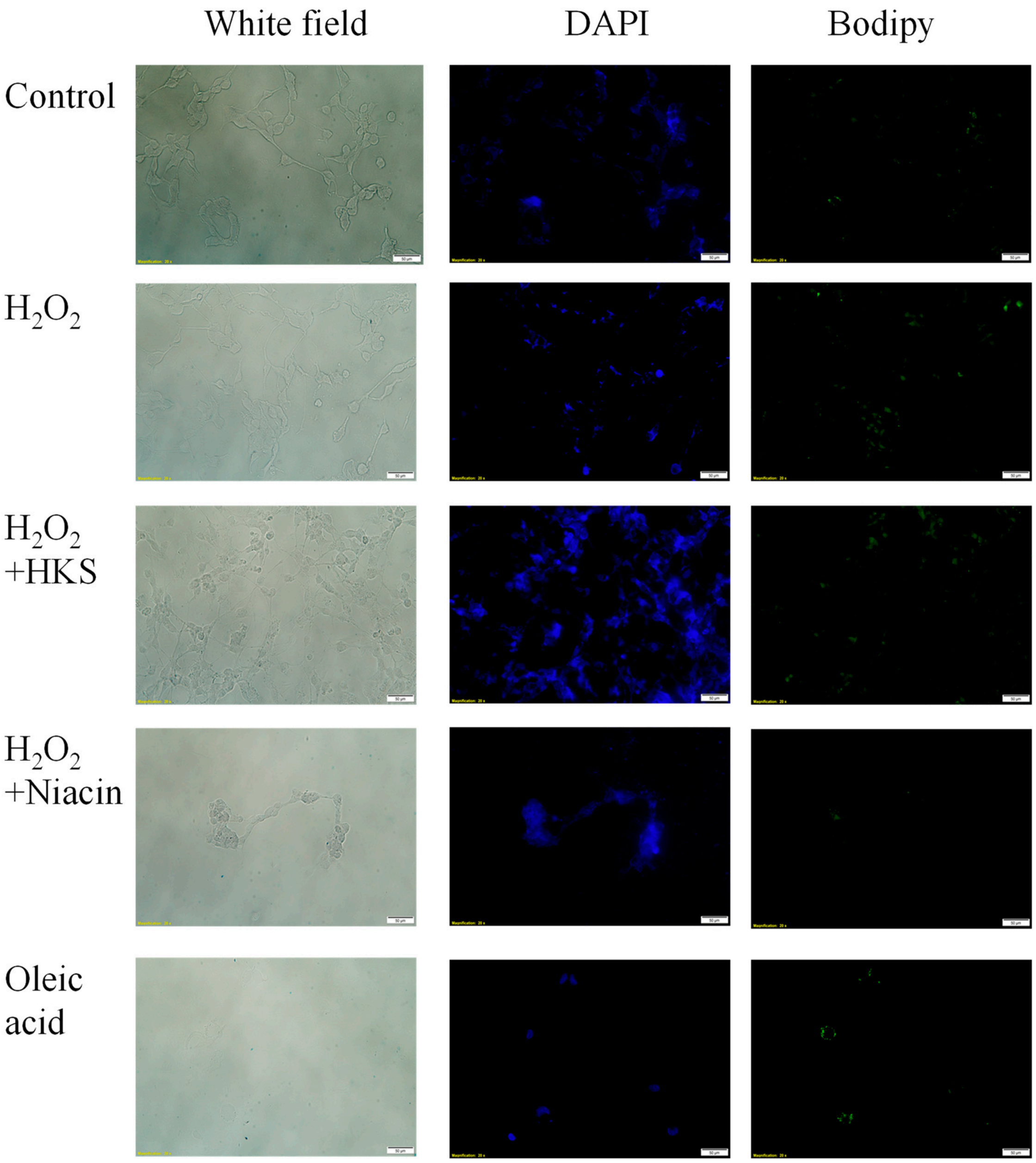

Supplementary Figure S1. Visualization of lipid droplets.

SH-SY5Y cells were exposed for 24 h to HKS (*B. breve* MCC1274 heat-killed cell extracts at 1% final v/v) or niacin (100 nM), with or without (PBS only as the control) H_2O_2 (200 μM) and then stained with DAPI or bodipy (2 μM). Oleic acid was used as a control to confirm the induction of lipid droplets (60 μM).
